# Casticin as potential anticancer agent: recent advancements in multi-mechanistic approaches

**DOI:** 10.3389/fmolb.2023.1157558

**Published:** 2023-05-26

**Authors:** Katya Carbone, Fabio Gervasi, Latipa Kozhamzharova, Nazgul Altybaeva, Eda Sönmez Gürer, Javad Sharifi-Rad, Christophe Hano, Daniela Calina

**Affiliations:** ^1^ CREA—Research Centre for Olive, Fruit and Citrus Crops, Rome, Italy; ^2^ Department of Scientific Works and International Relations, International Taraz Innovative Institute Named After Sherkhan Murtaza, Taraz, Kazakhstan; ^3^ Department of Molecular Biology and Genetics, Al-Farabi Kazakh National University, Al-frabi, Kazakhstan; ^4^ Department of Pharmacognosy, Faculty of Pharmacy, Sivas Cumhuriyet University, Sivas, Türkiye; ^5^ Facultad de Medicina, Universidad del Azuay, Cuenca, Ecuador; ^6^ Department of Biological Chemistry, Université ď Orléans, Orléans, France; ^7^ Department of Clinical Pharmacy, University of Medicine and Pharmacy of Craiova, Craiova, Romania

**Keywords:** casticin, *Vitex* spp., cancer, molecular mechanisms, signaling pathways, apoptosis

## Abstract

Plants, with their range of pharmacologically active molecules, represent the most promising source for the production of new anticancer drugs and for the formulation of adjuvants in chemotherapy treatments to reduce drug content and/or counteract the side effects of chemotherapy. Casticin is a major bioactive flavonoid isolated from several plants, mainly from the *Vitex* species. This compound is well known for its anti-inflammatory and antioxidant properties, which are mainly exploited in traditional medicine. Recently, the antineoplastic potential of casticin has attracted the attention of the scientific community for its ability to target multiple cancer pathways. The purpose of this review is, therefore, to present and critically analyze the antineoplastic potential of casticin, highlighting the molecular pathways underlying its antitumor effects. Bibliometric data were extracted from the Scopus database using the search strings “casticin” and “cancer” and analyzed using VOSviewer software to generate network maps to visualize the results. Overall, more than 50% of the articles were published since 2018 and even more recent studies have expanded the knowledge of casticin’s antitumor activity by adding interesting new mechanisms of action as a topoisomerase IIα inhibitor, DNA methylase 1 inhibitor, and an upregulator of the onco-suppressive miR-338-3p. Casticin counteracts cancer progression through the induction of apoptosis, cell cycle arrest, and metastasis arrest, acting on several pathways that are generally dysregulated in different types of cancer. In addition, they highlight that casticin can be considered as a promising epigenetic drug candidate to target not only cancer cells but also cancer stem-like cells.

## 1 Introduction

According to the World Cancer Research Fund International, 18,094,716 million cases of cancer were diagnosed worldwide in 2020, with a higher incidence in men compared to women (https://www.wcrf.org). Among the various types, breast and lung cancer were the most common cancers worldwide in 2020, contributing 12.5% and 12.2%, respectively, of the total number of new cases diagnosed during the year, followed by colorectal cancer with 1.9 million new cases in 2020, accounting for 10.7% of new cases (GBD, 2019 Colorectal Cancer Collaborators, 2022). According to the International Agency for Research on Cancer (IARC; accessed on 27 December 2022; https://gco.iarc.fr), the first two types of cancer also have the highest mortality rates worldwide. Moreover, according to the American National Cancer Institute (https://www.cancer.gov), by 2040, the number of new cancer cases per year is expected to rise to 29.5 million and the number of cancer-related deaths to 16.4 million. Treatment usually includes surgery, radiotherapy, and/or systemic therapy (chemotherapy, hormone treatments, and targeted biological therapies), depending on the type of tumor and the patient. Unfortunately, however, especially in the case of chemotherapy, which is still one of the most widely used therapies, there are considerable side effects from which the patient suffers (e.g., myelotoxicity, cardiotoxicity, renal, and pulmonary toxicity), which could be reduced by resorting in some cases to natural therapies, such as the use of products of plant origin with anticarcinogenic potential. Plants, with their array of secondary metabolites, represent an enormous potential to produce both new anticancer drugs and for the formulation of adjuvants in chemotherapy treatment to reduce drug content and/or counteract the side effects of chemotherapy (Iacopetta et al., 2017). Several anticancer agents of plant origin are currently on the market, such as vinca alkaloids, epipodophyllotoxins, taxanes, and camptotein derivatives. Among phytochemicals, flavonoids and polyphenolic compounds, which include isoflavonoids, flavanones, flavanols, flavonols, flavones, and anthocyanidins are increasingly indicated as potential anticarcinogenic agents capable of inducing apoptosis in malignant cells. Recently, studies in the literature have highlighted the antiproliferative and apoptotic potential of a polyphenolic compound of *Vitex* species: casticin (Chan et al., 2018; Ramchandani et al., 2020) (Supplementary Table S2). This compound has been used since ancient times and also in many traditional medicines, such as Unani, Ayurveda, Siddha, Chinese, and Roman, to treat different types of ailments, mainly female health issues, while recent studies have highlighted its antineoplastic potential and ability to enhance efficacy in combination with chemotherapeutic drugs (Rasul et al., 2014; Husna et al., 2021; Kamal et al., 2022).

Based on the pharmacological evidence related to casticin’s *in vitro* and *in vivo* activities, it has been demonstrated that it is a bioactive compound against several types of cancer such as bladder cancer (Chung and Kim, 2016; Huang et al., 2019; Xu et al., 2022), breast cancer (Song et al., 2010; Liu L. P. et al., 2014; Fan et al., 2018; Lang et al., 2019; Lang et al., 2020), cervical cancer (Xie et al., 2011; Zeng et al., 2012; Wang et al., 2022), colon cancer (Tang S. et al., 2013; Tang S. Y. et al., 2013; Qu et al., 2014; Shang et al., 2017; Kowalski et al., 2022), esophageal cancer (Song et al., 2016; Qiao et al., 2019), gallbladder cancer (Song et al., 2017), gastric cancer (Zhou et al., 2013b; Yang et al., 2017), glioma (Feng et al., 2012; Liu et al., 2013), hepatocellular cancer (He et al., 2013; He et al., 2014; Gao and Chen, 2015; Li et al., 2020), leukemia (Díaz et al., 2003; Li et al., 2005; Shen et al., 2009; Righeschi et al., 2012; Lai et al., 2019; Cheng et al., 2020a; Yu et al., 2022), lung cancer (Zhou et al., 2013a; Liu F. et al., 2014; Liou and Huang, 2017; Gong et al., 2018; Lang et al., 2019; Cheng et al., 2020b; Fu et al., 2022), melanoma (Shih et al., 2016; Shiue et al., 2016; Shih et al., 2017), nasopharyngeal cancer (Liu et al., 2019), oral cancer (Chou et al., 2018; Xie et al., 2019; Shang et al., 2020), ovarian cancer (Jiang et al., 2013; Wang et al., 2016; Zhang et al., 2018), pancreatic cancer (Huang, 2017; Lang et al., 2019), and prostate cancer (Meng et al., 2012; Lin et al., 2019), which are characterized by multitarget cellular mechanisms on multiple molecular pathways of the tumor cell, thus tackling several hallmarks of cancer. Therefore, the purpose of this updated and comprehensive review was to evaluate the current research literature on casticin with respect to its anticarcinogenic potential.

## 2 Literature-based quantitative research on casticin in cancer

To gain a general overview and characterize the papers published until 10 December 2022 on the anticancer properties of casticin, a bibliometric analysis was performed by sequentially using Scopus (an online abstract and citation database) and VOSviewer software (v.1.6.18). A Scopus search was performed (search within article title, abstract, and keywords) using the keywords (Search documents): “casticin” AND “cancer.” The 103 retrieved documents were subsequently “cleaned” of papers that did not contain the searched keywords in the title, abstract, or author keywords; furthermore, the cleaning process also applied to retraction- or erratum-type documents. As a result of this manual inspection, 75 documents were collected and analyzed with the Scopus function “analyze search results” to first characterize the obtained bibliographical dataset (“documents by year,” “documents by subject area,” and “documents by country or territory”). The line chart “documents by year” (Supplementary Figure S1A, Supplementary Table S1) describes the trend of the number of publications by year and shows that the research field of “casticin” and “cancer” is new and has a lifespan of exactly 20 years from 2002 to 2022. It started with only one document in 2002, and until 2010, the number of papers fluctuated between one and zero per year. Starting in 2012, the topic expanded rapidly within a few years, reaching a maximum peak of nine papers in 2013. Since 2016, the number of published documents has steadily fluctuated between six and eight, confirming a continuous and recent research interest in the anticancer properties of casticin. The pie chart “documents by subject area” (Supplementary Figure S1B) clearly shows that up to now, most of the research efforts have been devoted to elucidating the molecular mechanisms of the antitumor activity of casticin as the largest subject area is represented by “biochemistry, genetics, and molecular biology” with 35.6% of the total publications (47 total documents). The second largest field of interest was “medicine” (28.0%—37 documents), followed by the third largest subject area, which is represented by “pharmacology, toxicology, and pharmaceutics” (15.9%—21 documents). It is worth noting that the subject area “agricultural and biological science” is a very little explored field (3.0%— four documents). It should be expanded in the future because it is the authors’ opinion that in the future, if the potential of casticin in cancer therapy is confirmed by clinical trials, it will be necessary to make further research efforts to investigate the best species and varieties with the highest content of casticin in their tissues and which of them will be easy to purify from and easy to cultivate on a large scale. Interestingly, the bar chart (Supplementary Figure S1C) shows that among the top ten countries by number of publications, seven were from Asia, as were the top five, and by far the largest was China, with a total of 40 documents indexed in Scopus. That is not surprising because casticin is extracted from several *Vitis* species, have have long been used in traditional Chinese medicine (Ramchandani et al., 2020).

**FIGURE 1:**
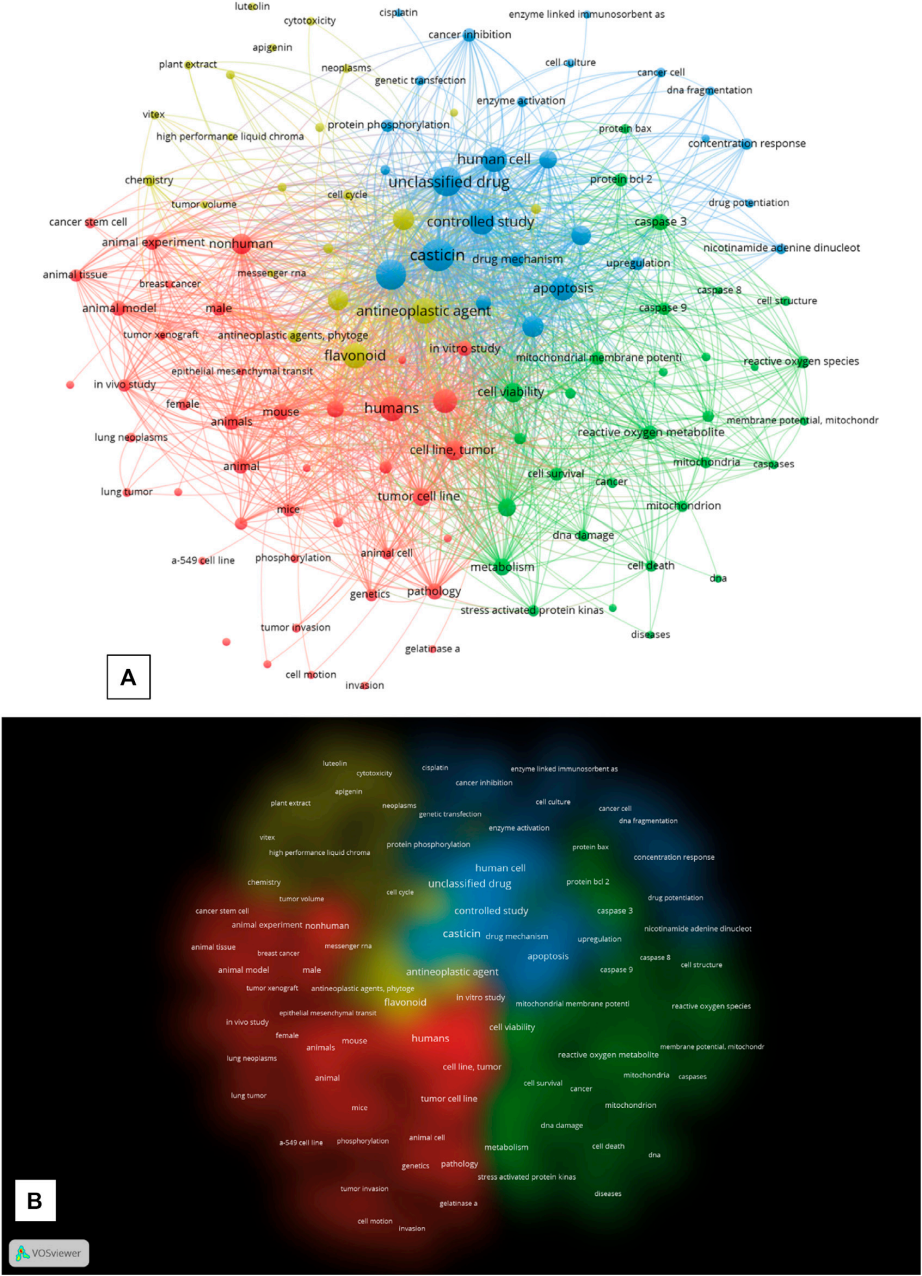
**(A)** “Network Visualization” of the keywords co-occurrence network map generated by VOS viewer. **(B)** “Density visualization”, “Cluster density” of the map generated in VOS viewer. Each density representing a cluster has a different colour. All the maps were generated by the VOSViewer software form the search results in SCOPUS (until 10^th^ of December 2022) after the query: “*casticin*” AND “*cancer*” and subsequent cleaning of the dataset.

The bibliographical dataset of 75 documents obtained from the cleaned Scopus query was further analyzed by calculating, plotting, and exporting the keyword distance network maps based on keyword co-occurrence from VOSviewer. Starting from this dataset, the software extracted 2,101 keywords, of which 183 met the default threshold of five minimum occurrences. The authors arbitrarily excluded the keywords “article,” “priority journal,” and “review” from the final calculation to make the topology of the network more relevant for the purpose of analysis. All other VOSviewer parameters were left at their default settings. The network visualization of VOSviewer shows the keyword co-occurrence network graph, with the keywords being represented by nodes and the co-occurrence in papers between two keywords by a connection (or link). The different diameters of the nodes represent the difference in the total number of occurrences of every single keyword extracted by the software from the dataset (the larger the diameter, the higher the number of occurrences). VOSviewer extracted four clusters (see Supplementary Table S1 for cluster keyword composition), each plotted with a different color in the network map and the cluster density map (Figures 1A, B).

The graph network visualization shows an overall general topology of highly interconnected nodes among the four clusters, which suggests a highly interconnected relationship of the keywords in the activity of casticin in cancer research, where the central node is represented by the keyword “casticin,” which scored the highest number of occurrences (70), being fully interconnected with nodes in all the four clusters. The first cluster (CS_1; red) is the largest and has “humans” as the most frequently occurring keyword (n. of occurrences: 43). It is composed of 42 keywords (nodes), which are related to research on the use of casticin in cancer both *in vitro* on tumor cell lines and *in vivo* on murine animal models, as suggested by the keywords “tumor cell line,” “mouse,” “animal model,” “*in vitro* study,” “mice,” “*in vivo* study,” and “tumor xenograft.” CS_2 (green) is composed of 30 nodes, among which the most frequently occurring keyword is “cell viability” (26 occurrences). It is composed of keywords extracted from publications of research studies on the casticin action mechanisms in cancer cells and particularly on their involvement in apoptosis signaling and mitochondrial physiology (“signal transduction,” “caspase 3,” “reactive oxygen species,” “mitochondrial membrane potential,” “protein bcl 2,” “caspase 9,” “mitochondria,” “protein bax,” “caspases,” “cell cycle arrest,” and “caspase 8”). CS_3 (light blue) is composed of 26 nodes, and the most frequently occurring keyword is “casticin,” which is the central node of the graph (70). It is composed of keywords that represent the research area of human cancer cell biology, and it is plotted between CS_2 and CS_4, with keywords representing a sort of “middle earth” between the two clusters (“human cell,” “apoptosis,” “protein expression,” “western blotting,” “flow cytometry,” “cancer inhibition,” “enzyme activation,” and “DNA fragmentation”). At the top border of the CS_3 node, “cisplatin” suggests that casticin was investigated not only alone but also in association with the chemotherapeutic antitumor agent cisplatin. CS_4 (yellow), in which the most frequently occurring keyword is “antineoplastic agent” with 45 total occurrences, is composed of 22 nodes representing the research performed to investigate the antineoplastic activity and the antiproliferative activity of casticin (“antineoplastic agent,” “antineoplastic activity,” “cell proliferation,” “antineoplastic agents,” “antiproliferative activity,” “cell cycle,” “cytotoxicity,” and “tumor volume”). Both the network visualization of keyword co-occurrences and the color cluster density maps (Figures 1A, B) show that all the clusters are not clearly separated, being at different degrees interspersed with each other. CS_4 has no sharp boundaries, and it is characterized by connections and overlaps with CS_1 and CS_3; CS_3 boundaries overlap with CS_2 and CS_4, also visually suggesting there is a sort of “middle earth” between the two. Overall, these observations confirm the strong linkage between all the studies carried out till today to investigate the use of casticin in cancer as a potential therapeutic agent, alone or in combination with other chemotherapeutic agents.

## 3 Phytochemistry

**FIGURE 2:**
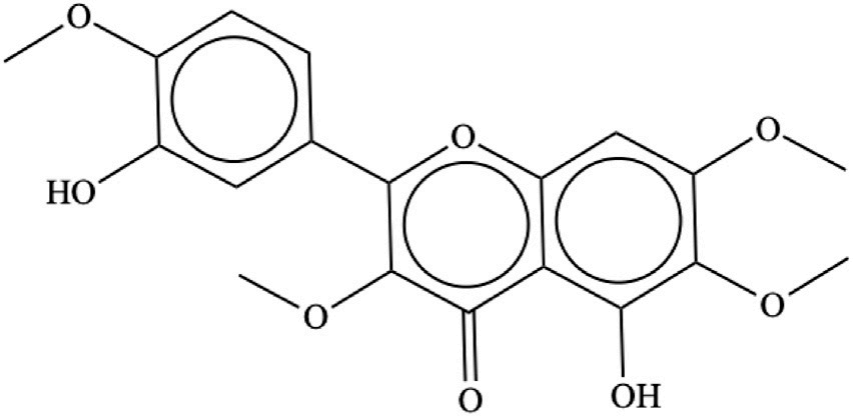
Casticin chemical structure.

Casticin [IUPAC name: 5-hydroxy-2-(3-hydroxy-4-methoxyphenyl)-3,6,7-trimethoxychromen-4-one; C_19_H_18_O_8_; CAS # 479-91-4; Figure 2], also known as vitexicarpin, is a tetramethoxyflavone with a molecular weight of 374.34. It is a solid with a melting point of 186°C–187°C and a solubility of 120.7 mg/L at 25°C. It is a flavonoid characteristic of *Vitex* spp., from which it has been isolated from leaves, fruits, and seeds. Its concentration in *V. agnus-castus* fruits varies from 0.03% to 1.18%, while in *V. trifolia* leaves, it is present at a concentration of 0.01% (Chan et al., 2018). It has also been isolated from other plants (Supplementary Table S2).

**FIGURE 3:**
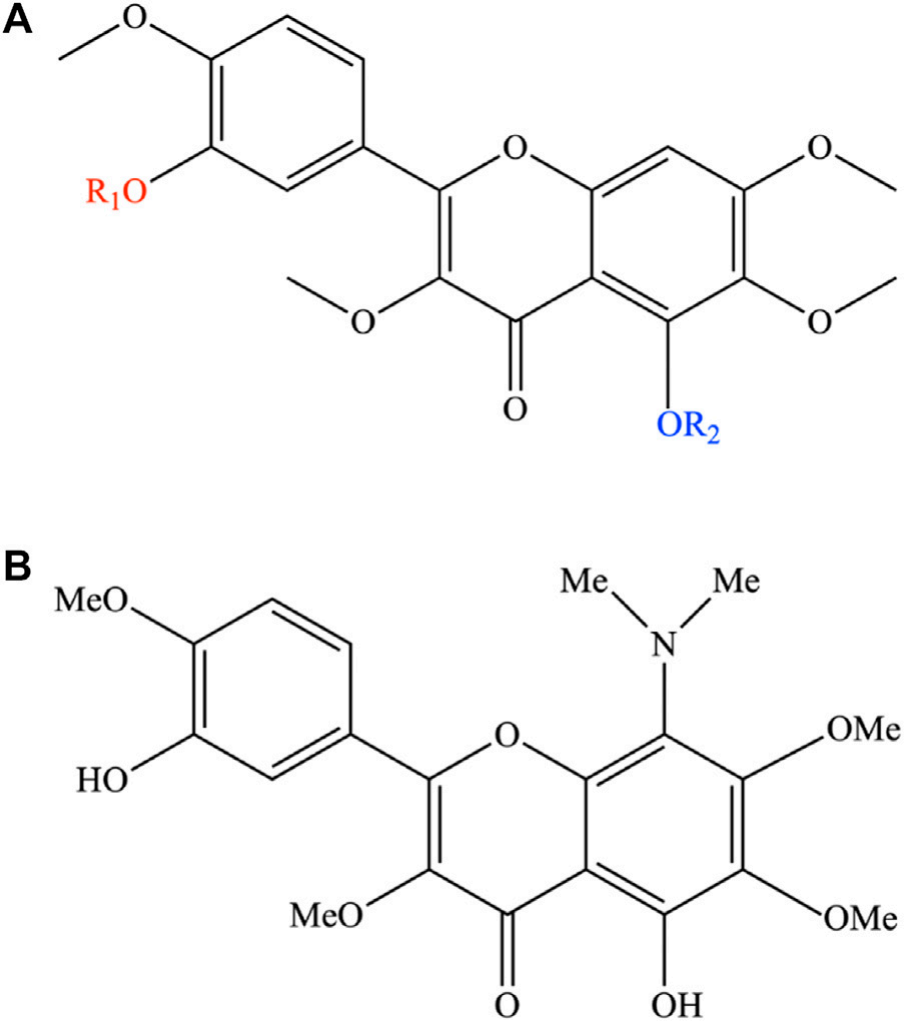
**(A)** Semi-synthetic derivates of casticin. In the figure, the functional groups coloured red and blue indicate the structural changes because of the synthesis reactions on the casticin. The semi-synthetic derivatives are obtained with the introduction of the following substituents: a) R_1_: -CH_3_; R_2_: -CH_3_. b) R_1_: C_6_H_5_O; R_2_: H. c) R_1_: C_6_H_5_O; R_2_: C_6_H_5_O. d) R_1_: CH_3_CO; R_2_: CH_3_CO. **(B)** Chemical structure of 8-dimethylaminocasticin.

Given the high bioactive potential of casticin, several attempts have been made to increase its cytotoxic potency by performing a series of acylation reactions on it to obtain a pool of semi-synthetic derivatives. While casticin was shown to be active toward a broad spectrum of malignant cells, the introduction of a methyl group (3,5,6,7,3′,4′-hexamethoxyflavone; substituent a; Figure 3) or one (3′-benzoyloxy-5-hydroxy-3,6,7,4′-tetramethoxyflavone; substituent b; Figure 3) or two benzoyl ones (5,3′-dibenzoyloxy-3,6,7,4′-tetramethoxyflavone; substituent c; Figure 3) resulted in complete inactivation or significant reduction in the cytotoxic activity (Díaz et al., 2003).

Moreover, the di-acetylated derivative (5,3′-diacetoxy-3,6,7,4′-tetramethoxyflavone; substituent d; Figure 3) showed a cytotoxicity profile like the original bioactive compound (Raffauf et al., 1981; Díaz et al., 2003). Lewin et al. (2010) showed that 8-dimethylaminocasticin (5,3′-dihydroxy-8-dimethylamino-3,6,7,4′-tetramethoxyflavone), a side-product obtained from the semi-synthesis reaction of 5,3′-dihydroxy-3,6,7,8,4′-pentamethoxyflavone, was weakly cytotoxic and completely inactive on the inhibition of tubulin polymerization, highlighting the presence of OH groups at C-5 and C-3′ and OCH_3_ groups at C-3 and C-4′ as favorable structural requirements for cytotoxicity and inhibition of ITP (Lewin et al., 2010).

## 4 Anticancer mechanisms of casticin

Cancer is a multifactorial, multi-stage disease involving complex cascades of signaling pathways, and despite the wide heterogeneity of the diverse human tumor types, most cancers share eight common characteristics known as “hallmarks of cancer,” which have been proposed to provide a conceptual scaffold to conceptualize the complex phenotypes of the diverse human cancer types in terms of a common set of underlying cellular parameters (Safarzadeh et al., 2014). These include sustained proliferative signaling, evasion of growth suppression, resistance to apoptosis, limitless proliferation, induction/access to the vasculature, activation of invasion and metastasis, reprogramming of cellular metabolism, and avoidance of immune destruction (Hanahan, 2022). The aim of this section is to highlight and discuss updates that have occurred in recent years about the most interesting studies that have shed light on new or confirmed mechanisms of the antitumor action of casticin.

**FIGURE 4:**
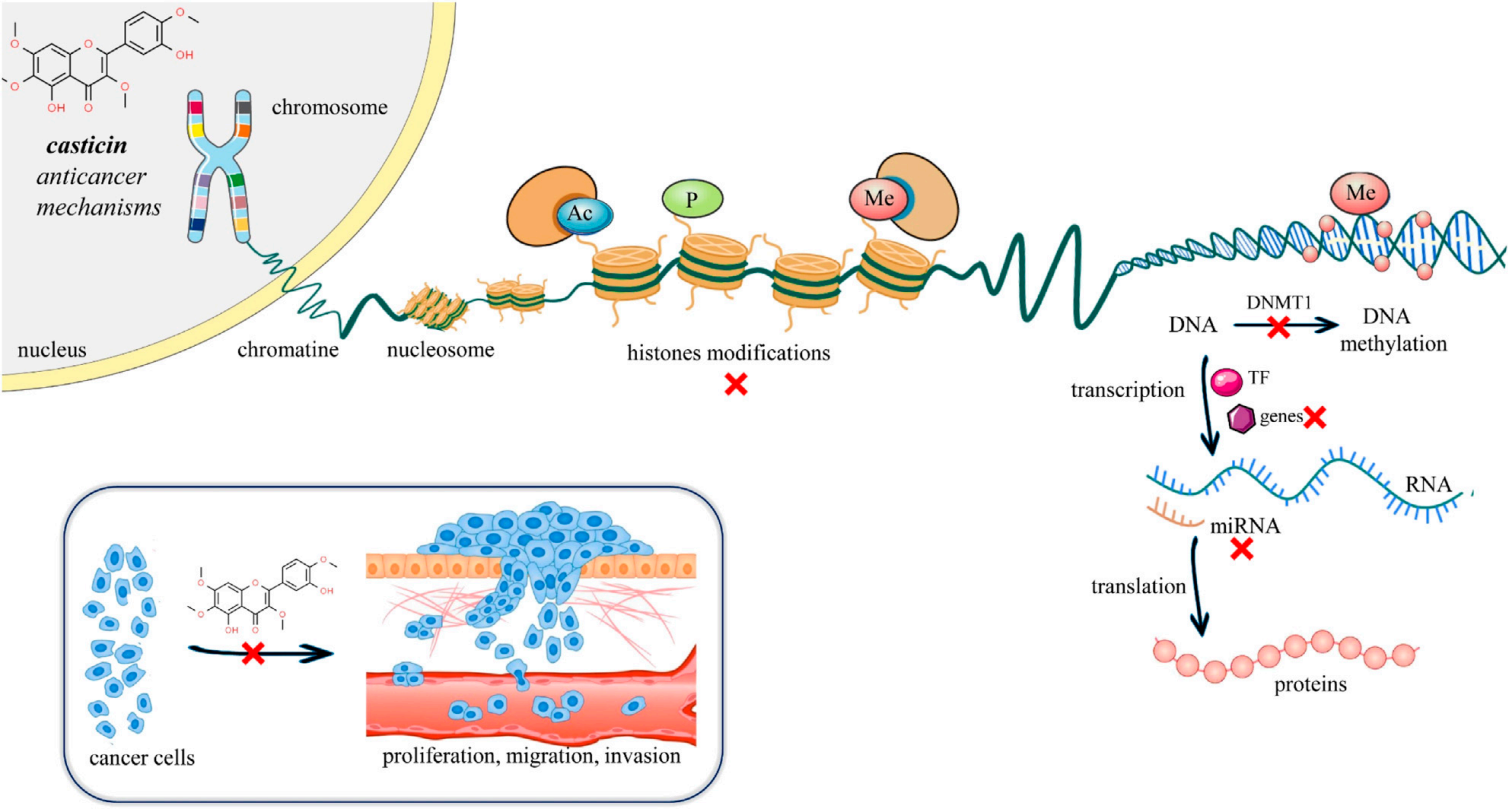
Illustrative scheme of mechanisms and epigenetic modulation of casticin in cancer. Recently, a growing body of evidence has highlighted a novel mechanism of casticin activity in gastric, cervical, and liver cancer cells, suggesting a possible use of casticin as a demethylation drug for the prevention and treatment of cancer (Yang et al., 2017; Li et al., 2020; Wang et al., 2022). Abbreviations and symbols: x, inhibition; Ac, acetylation; P, phosphorylation; Me, methylation; DNA, deoxyribonucleic acid; RNA, ribonucleic acid; and TF, transcription factor.

Cancer is considered a disease of the genome that manifests itself through complex mechanisms such as altered apoptosis, proliferative signals, or angiogenesis (Hanahan, 2022). Epigenetic interventions can induce such mechanisms without altering the gene sequence (Lee and Kim, 2022). Cancer is not an exclusively genetic disease. The progression of cancer depends on a multitude of other factors, such as the influence of the immune system, the tumor microenvironment, and external lifestyle factors. Epigenetic mechanisms can include either change in the interaction between DNA and proteins, changes in the conformation of the genome, or direct biochemical changes in the DNA molecule (Lee and Kim, 2022). The epigenome is the complete set of changes in DNA or DNA-associated proteins in a cell that alter gene expression but do not change the DNA sequence. Epigenetic changes, such as DNA methylation, have attracted increasing attention in studying tumor occurrence and progression with the aim of finding novel strategies for cancer treatment like epigenetic chemotherapy (Cheng et al., 2019) (Supplementary Table S3). DNA methylation has an important role in shaping chromatin structures, DNA conformation, DNA stability, and DNA–protein interactions. Abnormal methylation of DNA may inactivate tumor suppressor genes and activate oncogenes (Nishiyama and Nakanishi, 2021) (Figure 4).

Yang et al. (2017) by RT-qPCR and Western blotting clearly demonstrated that casticin increased the mRNA and protein levels of the tumor suppressor gene “reversion-inducing-cysteine-rich protein kazal (RECK) motif” in MGC803 gastric cancer cells in a dose-dependent manner, and such evidence was associated by the authors to a casticin-induced decrease of RECK promoter methylation levels by 31%, global cellular DNA methylation levels by 39%, and nuclear methylation activity by 71.6%. The decrease in intracellular methylation in MGC803 gastric cancer cells was supported by the author’s first report that casticin treatment drastically downregulated the mRNA and protein levels of “DNA methyltransferase 1” (DNMT1), whose gene family alteration causes aberrant methylation patterns responsible for tumor transformation and progression (Zhang and Xu, 2017) (Figure 4). The recently reported mechanism of casticin antitumor activity on the epigenome via the regulation of DNMT1 was shown very recently also in human cervical cancer stem-like cells (CCSLCs) via the regulation of both the enzyme activity and the expression (Wang et al., 2022). DNMT1 activity was found to increase compared with the non-CCSLC cell lines HeLa and CaSki. Furthermore, silencing DNMT1 in HeLa-derived CCSLCs lowered the stemness biomarkers CD133, CD44, Nanog, and Sox2 protein levels, whereas overexpression of DNMT1 showed the opposite effect. Most importantly, casticin treatment reduced DNMT1 enzyme activity, mRNA levels, and stemness in HeLa-derived CCSLCs. To confirm the hypothesis about the DNMT1 role in the casticin mechanism of action, DNMT1 silencing has been performed, and it enhanced the inhibitory effect of casticin on stemness; conversely, overexpression reversed the inhibitory effect of casticin on the stemness of HeLa cells. Taken together, these pieces of evidence show that casticin effectively inhibits stemness in CCSLCs by suppressing DNMT1 activation and expression, and as such, it is a promising epigenetic drug candidate to target not only cancer cells but also cancer stem-like cells, which are the most difficult cancer sub-population being characterized by recurrence, metastasis, heterogeneity, multidrug resistance, and radiation resistance (Yang et al., 2020).

The interplay between DNA methyltransferase 1 and microRNAs during tumorigenesis is well known (Yadav et al., 2021). Recent papers have pointed out that DNMT1 catalyzes the promoter methylation of miR-148a in pancreatic cancer (Hong et al., 2018) and acute myeloid leukemia (Wang et al., 2019); moreover, the aberrant expression of microRNAs is associated with tumor progression. Recent evidence showed that miR-148a-3p acts as a tumor-suppressive miRNA, which is downregulated in prostate cancer (Dybos et al., 2018), epithelial ovarian cancer (Wang et al., 2018), breast cancer (Chen et al., 2018), and hepatocellular carcinoma (Li et al., 2015; Jung et al., 2016). Another study confirmed the mechanism of inhibition of stemness in cancer cells (hepatocellular carcinoma) by casticin via DNMT1 suppression and evidenced for the first time the suppressive role that miRNA plays in the antitumor mechanism of casticin both *in vitro* and *in vivo* (Li et al., 2020). The authors demonstrated that casticin reduced stemness characteristics in two lines of hepatocellular carcinoma (HCC), MHCC97H and SK-Hep-1, by reducing the CD44^+^ cell population, the CD44 protein levels, and the expression of stemness biomarkers EpCAM, Bmi, Nanog, and Oct4 mRNAs. Also, casticin treatment decreased the enzyme activity and expression of DNMT1 and upregulated miR-148a-3p expression in HCC cell lines. To further demonstrate that casticin inhibits stemness characteristics by inhibiting DNMT1, the authors altered the expression of DNMT1 by using a lentiviral delivery system. They found that forced upregulation of DNA methyltransferase 1 enhanced the enzyme activity, neutralized the suppression of casticin on the activity of DNMT1, downregulated miR-148a-3p, and abrogated the upregulation of casticin on the expression of miR-148a-3p. Furthermore, forced upregulation of DNMT1 increased the CD44^+^ cell population, the CD44 protein levels, and the mRNA expression of the biomarkers EpCAM, Bmi, Nanog, and Oct4 and abrogated the inhibition of casticin on them. These results indicated that casticin inhibited the stemness characteristics of HCCs by inhibiting DNMT1.

In order to determine the role of miR-148a-3p in casticin-mediated repression of DNMT1 activity and stemness characteristics of HCC, miR-148a-3p mimic was transfected into MHCC97H and SK-Hep-1 cells. The results clearly showed that the transfected cells with the mimic significantly increased miR-148-3p in HCC cells, enhanced the repression of casticin on the activity of DNMT1, and reduced the stemness biomarkers. To finally validate the negative reciprocal regulation between DNMT1 and miR-148a-3p in the MHCC97H cell line *in vitro*, the authors showed that stable transfection of the DNMT1 gene with the lentiviral vector augmented the promoter methylation of miR-148a-3p, thus reducing its expression. Conversely, the luciferase reporter assay confirmed that miR-148a-3p specifically binds to the 3′-UTR wt but not to the 3′-UTR mutated version of DNMT1 mRNA, leading to methylase suppression. The authors also confirmed the role of casticin and miRNA *in vivo* and validated the *in vitro* findings.

Nude mice xenografted with MHCC97H tumor cells were treated with casticin (gavage), the mimic agonist agomir (synthetic)-148a-3p (intratumoral injection), casticin, and agomir-148a-3p (co-treatment). The results confirmed *in vivo* the significant reduction of xenograft tumor volume and weight in mice treated with casticin only, or agomir-148a-3p only, the reduction of DNMT1 activity and mRNA levels, and the reduction of the stemness biomarker CD44 expression. It is interesting to note that co-treatment of casticin and agomir-148a-3p showed the strongest inhibitory effect on the tumor weight and volume, the downregulation of DNMT1 and CD44 mRNAs, and the highest upregulation of miR-148a-3p. Taken together, these results showed that casticin has the ability to inhibit the stem cell-like phenotype of hepatocellular carcinoma by reversing the reciprocal negative regulation between DNMT1 and miR-148a-3p.

The role of miRNA in the casticin mechanism of antitumor action has also been reported in acute myeloid leukemia (AML) (Yu et al., 2022). The authors, among a set of previously reported aberrantly expressed miRNA in AML patients (Khalaj et al., 2014; Yeh et al., 2016; Fu et al., 2019), identified miR-338-3p only as being casticin-responsive. In fact, it is suppressed in two AML cell lines (HL60 and THP-1) compared to the normal bone marrow cells (HS-5), but it is upregulated in a dose-dependent manner in the AML cells after casticin treatment. To unravel the role of miR-338-3p in the casticin-induced HL60 cells, transfection of the miR-338-3p inhibitor was used to demonstrate that miR-338-3p inhibition abrogates the casticin-induced apoptosis and mitigates the casticin effects of inactivation on the PI3K/Akt signaling pathway; furthermore, the miR-338-3p knockdown made the casticin treatment non-effective. The authors, by luciferase reporter assay, experimentally validated the *in silico* predicted mRNA target of miR-338-3p: RUNX2, whose mRNA has a conserved binding site in the 3′-UTR for it. The involvement of RUNX2 in PI3K/Akt signaling is well-known in myeloid malignancies (Khalaj et al., 2014), and the authors demonstrated that miR-338-3p significantly suppressed both RUNX2 mRNA and protein. Moreover, casticin or miR-338-3p mimic showed similar activity in the reduction of the phosphorylation levels of PI3K (p-PI3K) and Akt (p-Akt), leading to pathway inactivation and casticin-induced growth arrest and apoptosis, which were instead increased by the miR-338-3p inhibitor, resulting in pathway activation via PI3K and Akt protein de-phosphorylation. The casticin inhibition of AML growth by promoting miR-338-3p expression was also confirmed *in vivo* in the mouse xenograft model. The miR-338-3p inhibitor abrogated the inhibitory effect of casticin on tumor volume and weight, the downregulation of RUNX2, and reduced p-PI3K and p-Akt levels, thus confirming the pivotal role of miR-338-3p in the casticin mechanism of action against acute myeloid leukemia. This growing body of evidence sheds light on a second new mechanism of casticin antitumor action by miRNA that has translational medical significance for the further development of casticin for cancer treatment.

Another mechanism of antitumor action of casticin has been shown very recently by Fu et al. (2022), supporting the first-time evidence that casticin also acts as an inhibitor of topoisomerase IIα in human non-small-cell lung cancer (NSCLC) cells. The gene TOP2A, encoding topoisomerase IIα or topo IIα proteins, is involved in the regulation of DNA replication, gene transcription, and DNA topology in cells (Nuncia-Cantarero et al., 2018). Acting as an oncogene, topo IIα is highly expressed in several cancers and is involved in the proliferation, migration, invasion, and other malignant characteristics of tumors (Ma et al., 2019). Moreover, previous studies have reported the expression of TOP2A as being regulated in lung cancer and lung cancer cell lines (Kou et al., 2020). The authors performed a global differential expression analysis by RNA-Seq in the non-small-cell lung cancer line A549 treated by casticin. GO functional enrichment analysis of differentially expressed genes (DEGs) described the well-known effects of casticin, which reduced the survival of A549 cells and induced apoptosis. Enrichment function classification included, among others, “DNA replications,” “DNA repair,” “cellular response to DNA damage stimulus,” and of KEGG signaling pathway “cell cycle,” “p53 signaling pathway”, “DNA replication”, and “FOXO signaling pathway.” The DEG analysis showed that TOP2A expression is repressed by casticin, and RT-qPCR confirmed such a pattern of expression; also, casticin induced double-strand breaks in DNA via TOP2A downregulation. These results were also validated in a different NSCLC cell line (H1299). Most interestingly, the authors performed an *in silico* molecular-docking analysis, which predicted the potential antitumor drug activity of casticin as an inhibitor by binding to topoisomerase IIα and so inducing apoptosis of cancer cells by DNA damage. Taken together, all the papers of the last 3 years widened the knowledge on casticin antitumor activity and added interesting new mechanisms of action as a topoisomerase IIα inhibitor, DNA methylase 1 inhibitor, and an upregulator of the onco-suppressive miR-338-3p, thus reinforcing the knowledge about its characteristic of being a multitarget drug that makes it a very promising anticancer medicine to be used alone or as an associative agent for combinative chemotherapy.

## 5 Limitations of casticin as a potential anticancer agent

An important therapeutic limitation related to the use of casticin as an individual anticancer agent is represented by insufficient bioavailability data in the human body. Also, its interactions with other natural bioactive compounds, which may reduce or enhance its therapeutic effectiveness, are not known. Therefore, specifically designed combinations of casticin or its combinations with other natural agents against defined tumor targets will extend the potential anticancer effects of such mixtures in controlled and reproducible ways. Another limitation of casticin as an anticancer agent is represented by the lack of human clinical trials and preclinical translational studies to accurately determine the effective anticancer therapeutic dose of casticin in humans.

## 6 Conclusion and future perspectives

Oncological diseases are one of the main causes of death in the modern world. The chemotherapeutic drugs used have severe adverse reactions, such as anemia, nausea, vomiting, headaches, and hair loss. Often, the side effects are so strong that the patient cannot tolerate the treatment. Therefore, the identification of safer and more effective therapies with selective action directed only at malignant cells is a hot topic of research in the field of oncological drugs. Integrated with oncological treatments, scientific phytotherapy supports therapeutic actions and offers support in reducing secondary reactions. It also offers the possibility of personalizing therapies and maintains a good quality of life for patients. Casticin showed a promising antitumor action due to its multitarget characteristic and high selectivity in cancer tissues, which are investigated both at the cellular level (anti-proliferation and cell cycle arrest in a time and dose-dependent manner, induction of apoptosis, angiogenesis inhibition, elimination of drug resistance, and anti-inflammatory) and the molecular level on different signaling pathways (PI3K/Akt, NF-kB, STAT3, FOXO3a/FoxM1, caspases, and Bax/Bcl-2, Akt/mTOR, ROS and Ca^2+^ upregulation, and hedgehog signaling). Further preclinical studies are needed to identify more specific molecular anticancer mechanisms of action and derived targets to fully understand the antitumor mechanisms and casticin as a potential therapeutic agent for the prevention and treatment of various cancers.

## Abbreviations

Ac, acetylation; AML, acute myeloid leukemia; DNA, deoxyribonucleic acid; DEGs, differentially expressed genes; DNMT1, DNA methyltransferase 1; EpCAM, epithelial cell adhesion molecule; FOXO, forkhead box transcription factors; Me, methylation; mRNA, messenger RNA; miRNA, microRNA; NANOG, homeobox protein; NSCLC, human non-small-cell lung cancer cells; P, phosphorylation; PI3K/Akt, phosphatidylinositol 3-kinase/protein kinase B; RNA, ribonucleic acid; RECK, reversion-inducing cysteine-rich protein kazal motif; RT-qPCR, reverse transcription-quantitative polymerase chain reaction; RUNX2, runt-related transcription factor 2; TF, transcription factor; and TOP2A, topoisomerase type IIA.
