# Supplementary material for: Casticin as potential anticancer agent: recent advancements in multi-mechanistic approaches
Source: Front Mol Biosci. 2023 May 26;10:1157558. doi: 10.3389/fmolb.2023.1157558 (PMC10250667; doi:10.3389/fmolb.2023.1157558)
Supplement: Supplementary file 1 [file Table1.docx]

**Supplementary Figures and Tables**


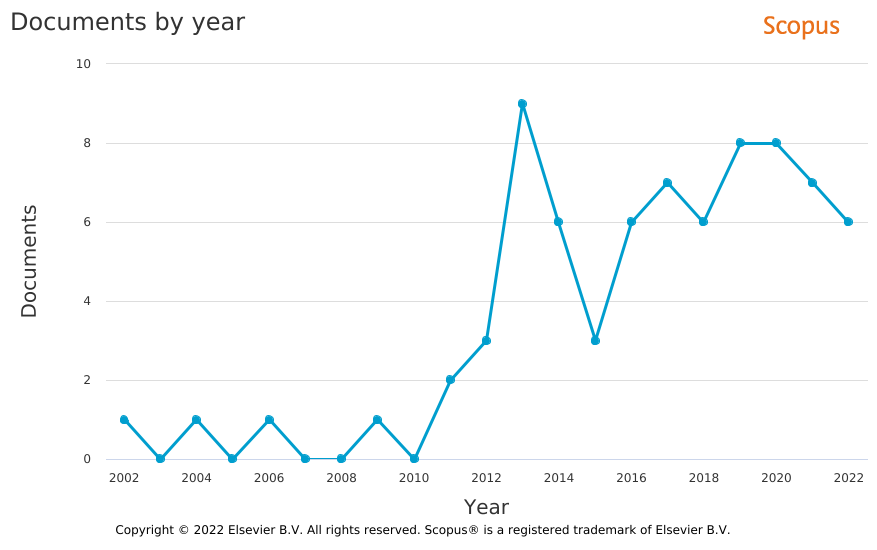


**(A)
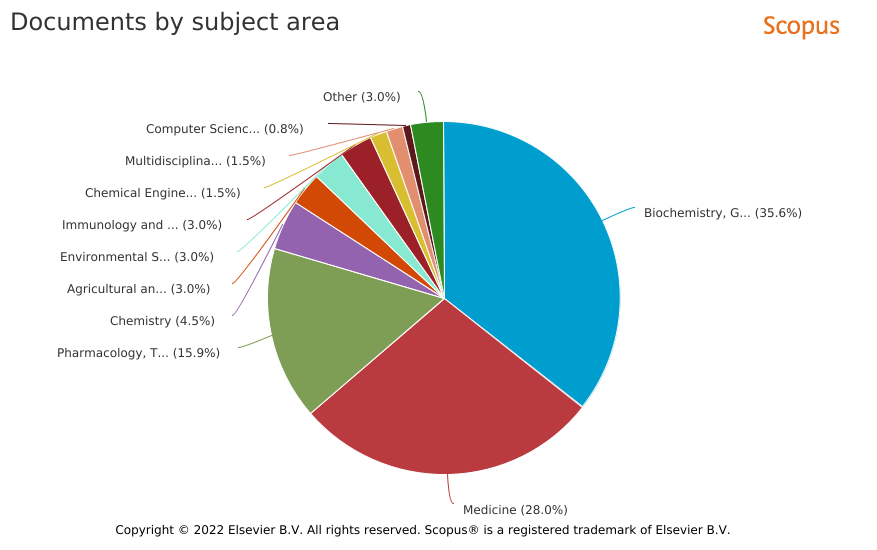
**

**(B)**

**
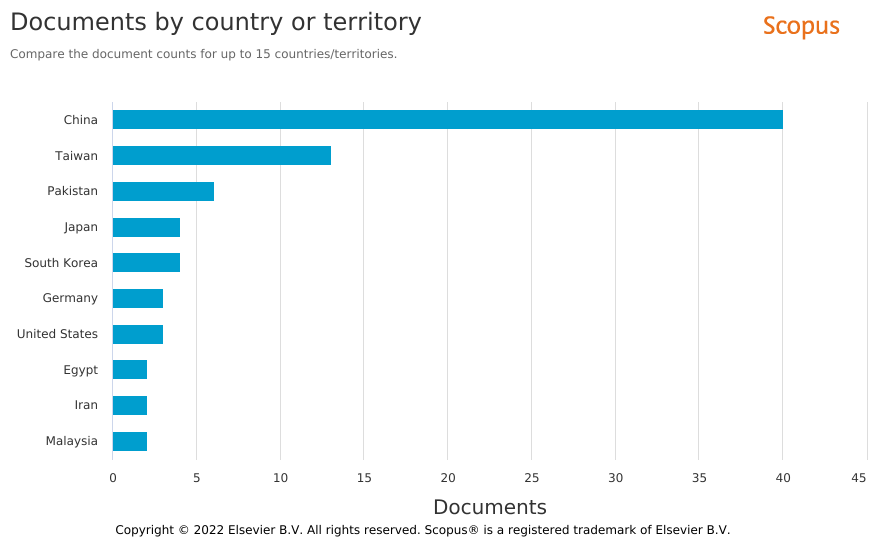
**

**(C)**

**Figure S1. (A)** Trends in number of published documents per year (“Documents by year”). **(B)** Pie graph of the percentage of documents classified by subject area (“Documents by subject area”). **(C)** Bar chart of top 10 countries in terms of number of published documents (“Documents by country or territory”). Statistics of quantitative distribution of published documents in SCOPUS after the query (performed on 10^th^ of December 2022): “*casticin*” AND “*cancer*” and subsequent cleaning of the dataset. All the charts were generated with the “Analyse search results” function.

| **Table S1.** Cluster composition of keyword co-occurrence network analysis from the cleaned dataset after SCOPUS search: “casticin” AND “cancer”. Order of keyword listing in each cluster: descending in the total number of occurrences. | | |
| --- | --- | --- |
| **Cluster**  **Number**  **(colour)** | **Number of keywords** | **Keywords**  **(descending order of total number of occurrences)** |
| 1  (red) | 42 | humans; flavonoids; nonhuman; cell line, tumor; tumor cell line; drug effect; animals; mouse; pathology; animal; animal model; in vitro study; animal experiment; male; mice; animal tissue; flavone derivative; genetics; in vivo study; protein kinase b; animal cell; female; cancer stem cell; lung neoplasms; phosphatidylinositol 3 kinase; lung tumor; phosphorylation; tumor invasion; tumor xenograft; a-549 cell line; breast cancer; cancer growth; cell motion; cell movement; drug screening; epithelial mesenchymal transition; gelatinase a; gene expression regulation; gene expression regulation, neoplastic; invasion; neoplasm invasiveness; proto-oncogene proteins c-akt |
| 2  (green) | 30 | cell viability; signal transduction; metabolism; caspase 3; reactive oxygen; metabolite; cell survival; mitochondrial membrane potential; protein bcl 2; caspase 9; reactive oxygen species; antineoplastic agents; dna damage; cell death; enzyme activity; mitochondria; mitochondrion; stress activated protein kinase; cancer; gene expression; cell structure; protein bax; caspases; cell cycle arrest; g2 phase cell cycle checkpoint; membrane potential, mitochondrial; caspase 8; cytochrome c; diseases; dna; drug effects |
| 3  (light blue) | 26 | casticin; human; unclassified drug; controlled study; human cell; apoptosis; protein expression; western blotting; flow cytometry; down regulation; drug mechanism; upregulation; cancer inhibition; protein phosphorylation;  concentration response; enzyme activation; nicotinamide adenine dinucleotide adenosine diphosphate ribosyltransferase; cancer cell; drug structure; genetic transfection; cell culture; dna fragmentation; drug potentiation; cisplatin; enzyme linked immunosorbent assay; gene silencing |
| 4  (yellow) | 22 | antineoplastic agent; flavonoid; antineoplastic activity; cell proliferation; antineoplastic agents, phytogenic; chemistry; antiproliferative activity; cell cycle; protein p53; cytotoxicity; mtt assay; neoplasms; plant extract; plant extracts; real time polymerase chain reaction; vitex; apigenin; high performance liquid chromatography; luteolin; messenger rna; protein p21;  tumor volume |

**Table S2**. Casticin plant sources^1^

| Taxonomy | References |  |
| --- | --- | --- |
| *Vitex agnus-castus* | (Mesaik et al., 2009) | 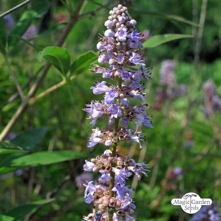 |
| *Vitex quinata* | (Li et al., 2010) | 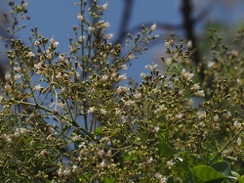 |
| *Vitex rotundifolia* | (Kobayakawa et al., 2004) | 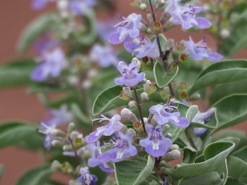 |
| *Artemisia annua* | (Shilin et al., 1989) | 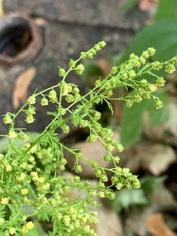 |
| *Tanacetum polycephalum^2^* | (Wollenweber and Rustaiyan, 1991) | 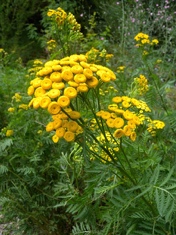 |
| *Callicarpa pilosissima* | (Chen et al., 2009) | 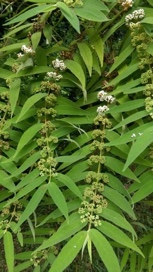 |
| *Lagophylla glandulosa* | (Bohm et al., 1992) | 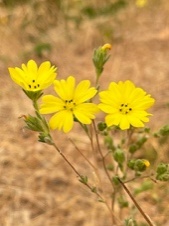 |
| *Achillea nobilis* | (Valant-Vetschera and Wollenweber, 1988a) | 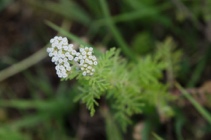 |
| *Achillea virescens* | (Valant-Vetschera and Wollenweber, 1988a) | 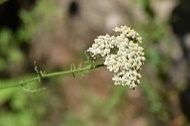 |
| *Chiliadenus montanus^3^* | (Ahmed et al., 1989) | 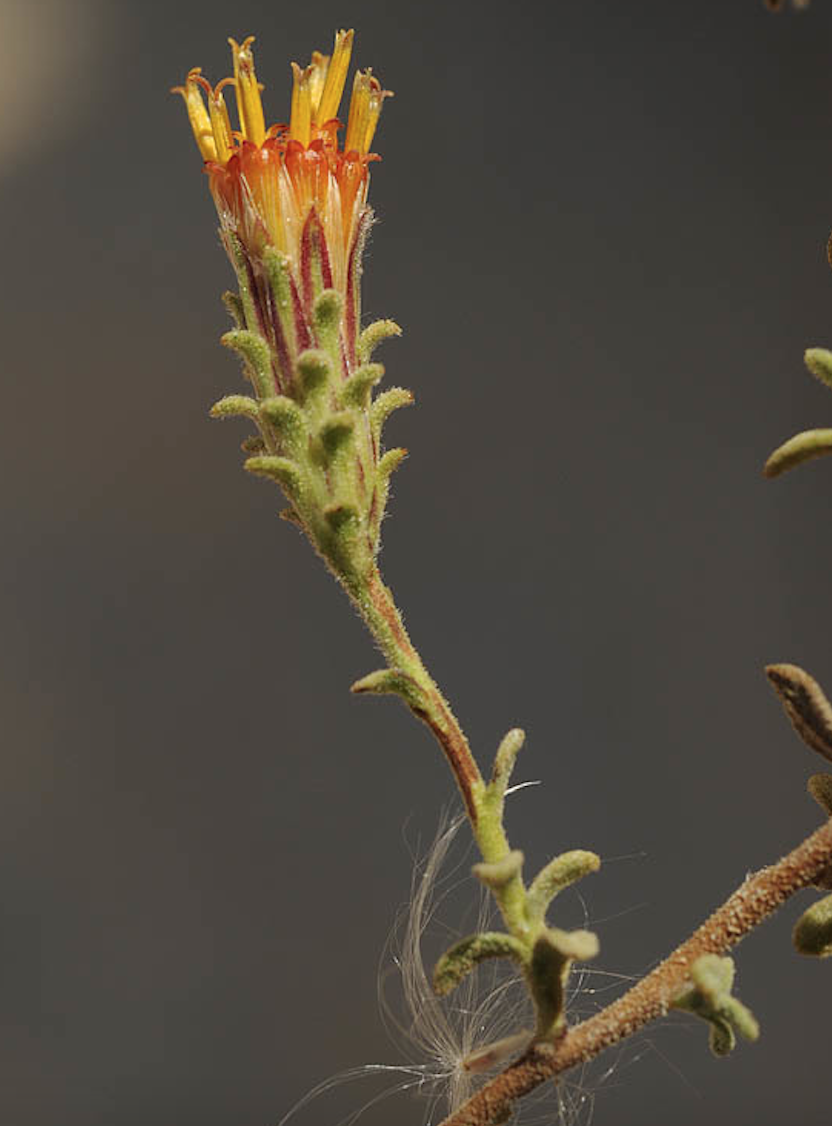 |
| *Artemisia scoparia* | (Brown et al., 2003) | 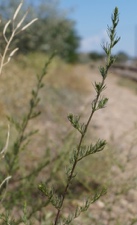 |
| *Achillea millefolium* | (Valant-Vetschera and Wollenweber, 1988b) | 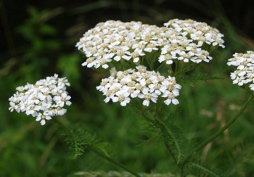 |
| *Achillea aspleniifolia* | (Valant-Vetschera and Wollenweber, 1988b) | 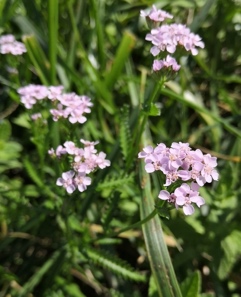 |
| *Achillea collina* | (Valant-Vetschera and Wollenweber, 1988b) | 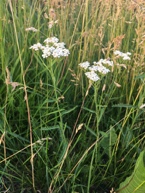 |
| *Artemisia judaica* | (Saleh et al., 1987) | 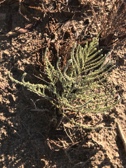 |
| *Brickellia baccharidea* | (Timmermann et al., 1981) | 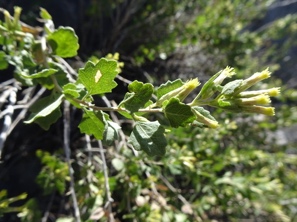 |
| *Artemisia carvifolia* | (Bergendorff and Sterner, 1995) | 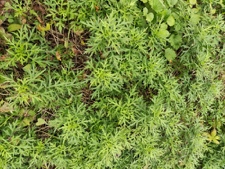 |
| *Artemisia apiacea^4^* | (Bergendorff and Sterner, 1995) | 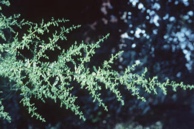 |
| *Artemisia abrotanum* | (Bergendorff and Sterner, 1995) | 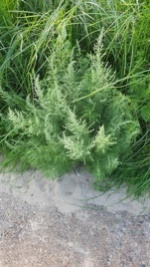 |
| *Bromelia pinguin* | (Raffauf et al., 1981) | 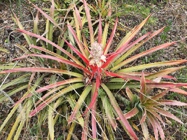 |
| *Artemisia alba* | (Barberá et al., 1986) | 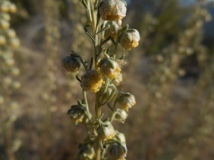 |
| *Artemisia incanescens* | (Barberá et al., 1986) | *Not found* |
| *Psiadia dentata* | (Jakobsen et al., 2001) | 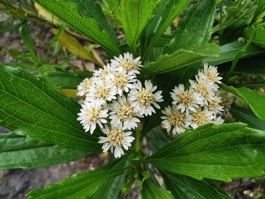 |
| *Achillea clavennae* | (Valant-Vetschera and Wollenweber, 1999) | 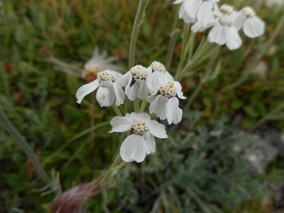 |
| *Achillea sibirica* | (Valant-Vetschera and Wollenweber, 1999) | 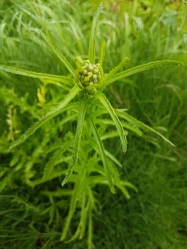 |
| *Achillea impatiens* | (Valant-Vetschera and Wollenweber, 1999) | 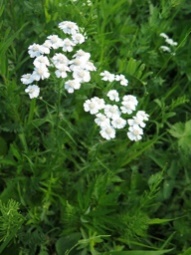 |
| *Achillea alpina* | (Valant-Vetschera and Wollenweber, 1999) | 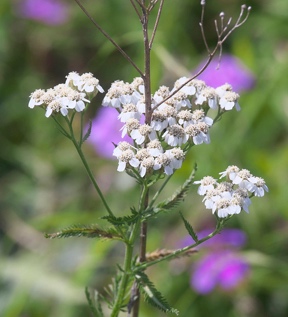 |
| *Vitex trifolia* | (Alam et al., 2002) | 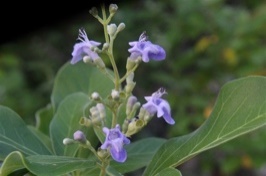 |
| *Psiadia viscosa^5^* | (Wang et al., 1989) | 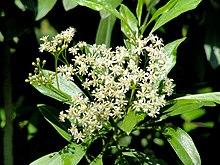 |
| *Artemisia capillaris* | (Brown et al., 2003) | 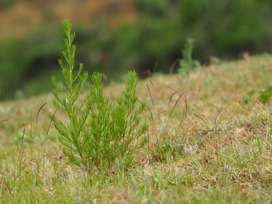 |
| *Waltheria indica* | (Cretton et al., 2016) | 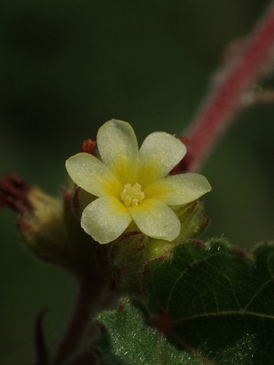 |
| *Laggera alata* | (Zheng et al., 2003) | 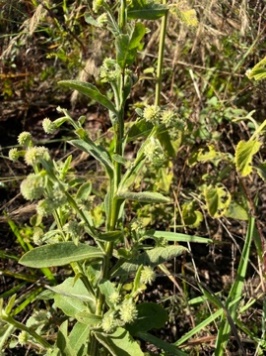 |
| *Tessaria integrifolia* | (Guerreiro et al., 1990) | 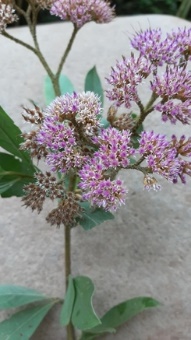 |
| *Vitex negundo* | (Díaz et al., 2003) | 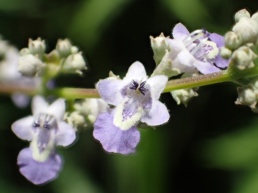 |
| *Parthenium ligulatum* | (Mears, 1973) | 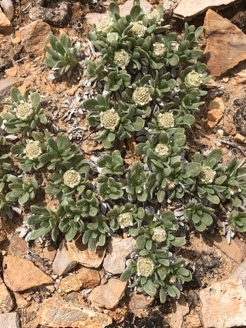 |
| *Eremophila mitchellii* | (Barnes et al., 2011) | 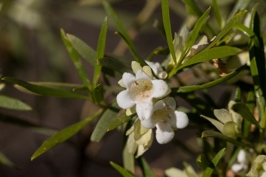 |
| *Coleus cylindraceus* | (Orabi et al., 2000) | 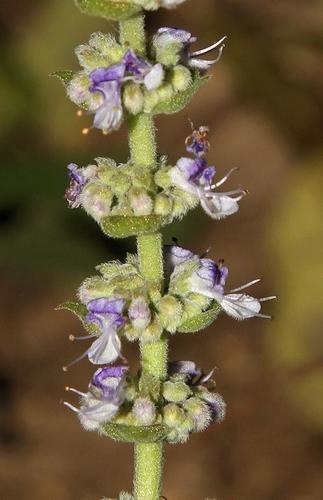 |
| *Plectranthus montanus* | (Orabi et al., 2000) | 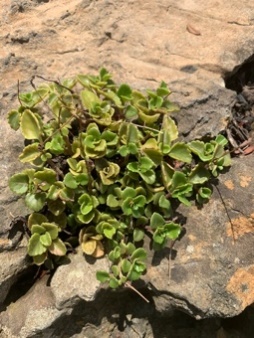 |
| *Helianthus microcephalus* | (Gao et al., 1987) | 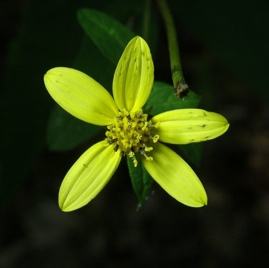 |
| *Pluchea sagittalis* | (Zani et al., 1994) | 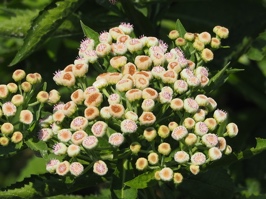 |
| *Artemisia pedemontana* | (Horie et al., 1989) | 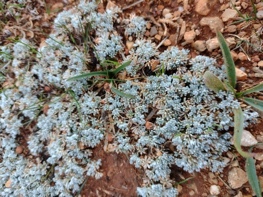 |
| *Artemisia nitida^6^* | (Horie et al., 1989) | 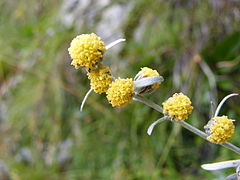 |

^1^adapted from National Center for Biotechnology Information (2023). PubChem Compound Summary for CID 5315263, Casticin. Retrieved December 27, 2022 from [https://pubchem.ncbi.nlm.nih.gov/compound/Casticin.](https://pubchem.ncbi.nlm.nih.gov/compound/Casticin.%202)

^2^source of plant image: https://commons.wikimedia.org/wiki/File:Tanacetum_vulgare_BOGA_1.jpg

^3^source of plant image: <https://eol.org/media/9297382>; licence: cc-by-nc-sa-3.0; creator: Ori Fragman-Sapir

^4^source of plant image: <http://www.epilepsynaturapedia.com/index.php/Artemisia_apiacea>

^5^source of plant image: <https://www.wikidata.org/wiki/Q15600975>

^6^source of plant image: <https://commons.wikimedia.org/w/index.php?search=Artemisia+nitida&title=Special:MediaSearch&go=Go&type=image>

Where not specifically indicated the source of plant images is: <https://www.inaturalist.org/> (accessed on December, 27^th^, 2022).

**Table S3**. Mechanism of action and molecular targets for casticin resulting in chemotherapeutic activity^1^

| Gene symbol | Up regulation | Biochemical function^2^ |  | Gene symbol | Down regulation | Biochemical function^2^ |
| --- | --- | --- | --- | --- | --- | --- |
| AIFM1 | expression of AIFM1 protein | Apoptotic/antiapoptotic genes |  | AKT3 | expression of AKT3 mRNA | Protein kinase |
| ALDH1A1 | expression of ALDH1A1 mRNA |  |  | ATM | expression of ATM protein | Protein kinase |
| APAF1 | expression of APAF1 protein | Apoptotic/antiapoptotic genes |  | ATR | expression of ATR protein | Protein kinase |
| ATF6 | expression of ATF6 protein | Trascriptional factors |  | BCL2 | expression of BCL2 protein | Apoptotic/antiapoptotic genes |
| AXIN2 | expression of AXIN2 mRNA | Protein coding gene |  | BCL2L1 | expression of BCL2L1 protein | Protein coding gene |
| BAK1 | expression of BAK1 protein | Apoptotic/antiapoptotic genes |  | BID | expression of BID protein | Protein coding gene |
| BAMBI | expression of BAMBI mRNA | Protein coding gene |  | BMI1 | expression of BMI 1 protein; expression of BMI1 mRNA | Protein coding gene |
| BAX | expression of BAX protein | Apoptotic/antiapoptotic genes |  | BNIP2 | expression of BNIP2 mRNA | Protein coding gene |
| BCL2L1 | expression of BCL2L1 mRNA | Apoptotic/antiapoptotic genes |  | CAMK4 | expression of CAMK4 mRNA | Protein coding gene |
| BID | expression of BID protein | Apoptotic/antiapoptotic genes |  | CCNB1 | expression of CCNB1 protein | Protein coding gene |
| BMP4 | expression of BMP4 mRNA | Growth factors and receptors |  | CD44 | expression of CD44 protein | Growth factors and receptors |
| BRCA1 | expression of BRCA1 protein | Cell cycle regulator |  | CD83 | expression of CD83 mRNA | Protein coding gene |
| CAPN1 | expression of and results in increased cleavage of CAPN1 protein | Enzyme and hormones |  | CDC25C | expression of CDC25C protein | Cell cycle regulator |
| CAPN2 | expression of CAPN2 protein | Enzyme and hormones |  | CDKN1A | expression of CDKN1A mRNA | Cell cycle regulator |
| CASP2 | expression of CASP2 protein | Apoptotic/antiapoptotic genes |  | CDKN1A | expression of CDKN1A protein | Cell cycle regulator |
| CASP3 | activity of CASP3 protein | Apoptotic/antiapoptotic genes |  | CLDN1 | expression of CLDN1 mRNA | Protein coding gene |
| CASP3 | cleavage of and results in increased activity of CASP3 protein | Apoptotic/antiapoptotic genes |  | COL1A1 | expression of COL1A1 mRNA | Cell cycle regulator |
| CASP4 | expression of CASP4 protein | Apoptotic/antiapoptotic genes |  | DKK3 | expression of DKK3 mRNA | Cell signalling |
| CASP6 | expression of CASP6 protein | Apoptotic/antiapoptotic genes |  | DNMT1 | activity of DNMT1 protein | Transcriptional factor |
| CASP7 | expression of CASP7 protein | Apoptotic/antiapoptotic genes |  | ENDOG | expression of ENDOG protein | Protein coding gene |
| CASP8 | expression of and results in increased activity of CASP8 protein | Apoptotic/antiapoptotic genes |  | EP300 | expression of EP300 mRNA | Transcriptional factor |
| CASP9 | activity of CASP9 protein | Apoptotic/antiapoptotic genes |  | EPCAM | expression of EPCAM mRNA | Protein coding gene |
| CD24 | expression of CD24 mRNA | Cell cycle regulator |  | FASLG | expression of FASLG protein | Apoptotic/antiapoptotic genes |
| CDH17 | expression of CDH17 mRNA | Apoptotic/antiapoptotic genes |  | FOXO3 | expression of FOXO3 mRNA | Transcriptional factor |
| CDK2 | expression of CDK2 protein | Cell cycle regulator |  | GRID2 | expression of GRID2 mRNA | Protein coding gene |
| CDKN1B | expression of CDKN1B mRNA | Cell cycle regulator |  | HCN2 | expression of HCN2 mRNA | Hyperpolarization-activated cation channel |
| CDKN2A | expression of CDKN2A protein | Cell cycle regulator |  | HOXB9 | expression of HOXB9 mRNA | Transcriptional factor |
| CHEK2 | expression of CHEK2 protein | Protein kinase |  | IL12A | expression of IL12A mRNA | Growth factors and receptors |
| CREB1 | expression of CREB1 mRNA | Transcriptional factor |  | IL6 | expression of IL6 mRNA | Growth factors and receptors |
| CYCS | expression of CYCS protein | Apoptotic/antiapoptotic genes |  | LDHB | expression of LDHB mRNA | Enzyme and hormones |
| ENC1 | expression of ENC1 mRNA | Process regulation |  | LEF1 | expression of LEF1 mRNA | Transcriptional factor |
| ERBB3 | expression of ERBB3 mRNA | Protein kinase |  | LPL | expression of LPL mRNA | Enzyme and hormones |
| FADD | expression of FADD protein | Apoptotic/antiapoptotic genes |  | MCL1 | expression of MCL1 protein | Apoptotic/antiapoptotic genes |
| FAS | expression of FAS protein | Growth factors and receptors |  | MIR148A | expression of MIR148A | Tumor suppressor |
| FASLG | expression of FASLG protein | Growth factors and receptors |  | MMP2 | expression of MMP2 mRNA | Enzyme and hormones |
| HSPA5 | expression of HSPA5 protein | Apoptotic/antiapoptotic genes |  | NANOG | expression of NANOG protein | Transcriptional factor |
| IRAK4 | expression of IRAK4 mRNA | Enzyme and hormones |  | NFKB1 | expression of NFKB1 mRNA | Transcriptional factor |
| LRP5 | expression of LRP5 mRNA | Developmental protein, Receptor |  | NFKB2 | expression of NFKB2 mRNA | Transcriptional factor |
| MAP3K7 | expression of MAP3K7 mRNA | Protein kinase |  | NFKBIA | expression of NFKBIA mRNA | Transcriptional factor |
| MDC1 | expression of MDC1 protein | Cell cycle regulator |  | NFKBIB | expression of NFKBIB mRNA | Transcriptional factor |
| MUC13 | expression of MUC13 mRNA | Cell signalling |  | NFKBID | expression of NFKBID mRNA | Transcriptional factor |
| MYD88 | expression of MYD88 mRNA | Cell signalling |  | NFKBIE | expression of NFKBIE mRNA | Transcriptional factor |
| NKD1 | expression of NKD1 mRNA | Cell signalling |  | NRG3 | expression of NRG3 mRNA | Growth factors and receptors |
| PPARG | expression of PPARG mRNA | Transcriptional factor |  | OLIG2 | expression of OLIG2 mRNA | Transcriptional factor |
| SMAD1 | expression of SMAD1 mRNA | Transcriptional factor |  | PAK3 | expression of PAK3 mRNA | Protein kinase |
| SMAD3 | expression of SMAD3 mRNA | Transcriptional factor |  | POU5F1 | expression of POU5F1 protein | Transcriptional factor |
| TMBIM4 | expression of TMBIM4 mRNA | Apoptotic/antiapoptotic genes |  | PPARD | expression of PPARD mRNA | Transcriptional factor |
| TMEM45B | expression of TMEM45B mRNA | Cell cycle regulator |  | PRKAR2B | expression of PRKAR2B mRNA | Protein kinase |
| TNFSF10 | expression of TNFSF10 protein | Apoptotic/antiapoptotic genes |  | PRKDC | expression of PRKDC protein | Enzyme and hormones |
| TRAP1 | expression of TRAP1 mRNA | Apoptotic/antiapoptotic genes |  | SPARC | expression of SPARC mRNA | Cell cycle regulator |
| XBP1 | expression of XBP1 mRNA | Transcriptional factor |  | TAB2 | expression of TAB2 mRNA | Protein kinase |
| YWHAB | expression of YWHAB protein | Cell signalling |  | TLR4 | expression of TLR4 mRNA | Cell cycle regulator |

^1^adapted from National Center for Biotechnology Information (2023). PubChem Compound Summary for CID 5315263, Casticin. Retrieved December 27, 2022 from https://pubchem.ncbi.nlm.nih.gov/compound/Casticin. ^2^adapted from <https://www.proteinatlas.org/>
